# Identification of plankton habitats in the North Sea

**DOI:** 10.1002/ece3.70342

**Published:** 2024-09-30

**Authors:** Rene‐Marcel Plonus, Jens Floeter

**Affiliations:** ^1^ Institute of Marine Ecosystem and Fishery Science University of Hamburg Hamburg Germany

**Keywords:** habitat maps, machine learning, North Sea, plankton distributions, plankton–habitat associations

## Abstract

The definition of an ecological niche makes it possible to anticipate the responses of a species to changing environmental conditions. Broad tolerance limits and a paucity of readily observable niches in the pelagic zone make it difficult to anticipate responses of the plankton community related to anthropogenic or environmental changes. Plankton distributions are closely linked to climate change and shape the seascape for higher trophic levels, so monitoring plankton distributions and defining ecological niches will help to understand and predict ecosystem responses. Here we apply a machine learning autoencoder and a density‐based clustering algorithm to high‐frequency datasets sampled with a ROTV Triaxus in the North Sea. The results indicate that in this highly dynamic environment, local hydrography prevents niche‐based separation of plankton species at the sub‐mesoscale, despite the availability of different habitats. Plankton patches were associated with naturally occurring frontal systems and anthropogenically induced upwelling‐downwelling dipoles in the vicinity of offshore wind farms (OWFs).

## Graphical abstract

The definition of pelagic habitats and ecological niches is a critical prerequisite for the successful management of the marine ecosystem. Here we propose a fully automatic method to monitor and quantify pelagic habitats in the southern North Sea based on physical and biological data alike.

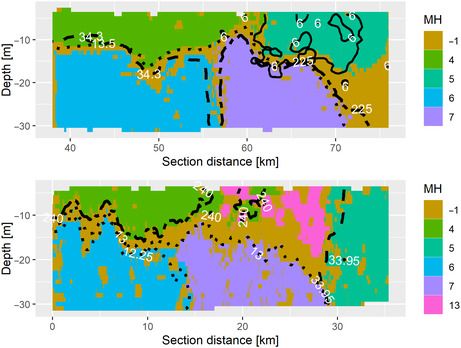

## 1 INTRODUCTION

The concept of an “ecological niche” was first applied by Grinnell (1917) and referred to the abiotic demands of a species toward its environment and the behavioral adaptations of the species to the same (Grinnellian niche). A formal definition of the concept followed in Hutchinson (1957), describing an ecological niche as an ‘n‐dimensional hypervolume’, where each dimension is influenced by a different environmental parameter. A further specification by Hutchinson (1957) was the distinction between fundamental and realized ecological niches. While the fundamental niche represents the total area that allows a population to survive, the realized niche describes the area where a species dominates over competitors. The latter refers more to the “Eltonian niche”, which focuses on the interaction of different species (Elton, 1927), especially in the more modern differentiation of the two (Dehling & Stouffer, 2018). The original understanding of the Eltonian niche as stated by Hutchinson (1957) assumes that co‐occurring species occupy different niches that do not intersect. The paucity of readily observable physical niches in the pelagic zone (Behrenfeld et al., 2021) and a seemingly unstructured environment (Martin et al., 2021) let to the formulation of the ‘paradox of plankton’ by Hutchinson (1961): the co‐existence of relatively many species in an apparently homogeneous environment, even though species richness tends to increase with habitat heterogeneity (Lapointe & Bourget, 1999; MacArthur & MacArthur, 1961). However, niche‐based models do not explain redundancy (Leibold & McPeek, 2006) or the existence of functional groups or traits (Barton et al., 2013; Dehling & Stouffer, 2018), both of which have been observed in plankton communities and are generally considered positive aspects of biodiversity (Leibold & McPeek, 2006).

The term “plankton” encompasses a diverse group of organisms in the oceans, including pytho‐ as well as zooplankton. Here we will use it to refer to the zooplankton component only. Traditional methods in plankton ecology have been time‐consuming and thus prevented the up‐scale to pan‐oceanic observations (Irisson et al., 2022). This paucity of data and the inconsistency in sampling methods and scales have limited our understanding of the factors and processes determining the abundance or diversity of plankton (Lombard et al., 2019). New optical sampling methods have emerged in the last decade which produce a wealth of information (Irisson et al., 2022), but their scientific use was limited by the concepts and methods applicable to the huge amount of data they generate (Alvarez‐Berastegui et al., 2014; Irisson et al., 2022; Lombard et al., 2019; North et al., 2016). In addition, the traditional view of turbulence‐homogenized plankton communities has been challenged by recent studies, suggesting that biological and physical processes create a structured realm at scales down to a few centimeters (Basterretxea et al., 2020; Mitchell et al., 2008).

One way to handle these data is habitat maps, which link bio‐physically distinct areas to specific species communities (Harris & Baker, 2012). However, due to the high variability of relevant spatial and temporal scales in the pelagic environment (Hinchey et al., 2008; Thompson et al., 2016) it is a daunting task to accurately determine pelagic habitats and identify associated plankton communities.

That is where machine learning excels. Several studies have recently created a link between global plankton datasets and associated physical variables (Busseni et al., 2020; Cael et al., 2021; Drago et al., 2022; Sonnewald et al., 2020). But even though machine learning has already successfully brought insights into plankton ecology in the past, fully automated predictions can still only be trusted for the most abundant species (Irisson et al., 2022; Plonus, Conradt, et al., 2021). Fortunately, plankton communities are usually highly diverse (Siegel, 1998) but dominated by a few, very abundant taxa (Fuhrman, 2009).

For example, there are 31 species known to have pluteus larvae, the larval form of echinoderms, in the North Sea (Laakmann et al., 2016). However, *Echinocardium cordatum* (Pennant, 1777) is the most abundant one, probably due to higher winter temperatures (Kirby et al., 2007), complemented by *Amphiura* spp. (Lindley et al., 1995). Since the 1980s, pluteus larvae have become even more abundant than copepods at times (Kirby et al., 2007; Lindley et al., 1995), which usually numerically dominate the zooplankton community. An extensive overview of copepod species in the North Sea was provided by Fransz et al. (1991). Even though there are several different species, four of them provide 85% of the biomass, namely *Acartia clausi* (Giesbrecht, 1889), *Centropages hamatus* (Liljeborg, 1853), *Temora longicornis* (Müller, 1785), and *Pseudocalanus elongatus* (Boeck, 1865) (Beaugrand et al., 2002; Hickel, 1975). The by far most abundant one is *P. elongatus* (Fransz et al., 1991). Second in abundance only to copepods (Landry et al., 1994), appendicularians are of special importance for the vertical particle fluxes in the world's oceans (Winder et al., 2017). Due to their affinity for higher temperatures and tolerance toward more acidic conditions, it is likely that their importance will increase under global warming (Winder et al., 2017). The most abundant species in European coastal waters are *Oikopleura longicauda* Vogt, 1854, *Oikopleura dioica* Fol, 1872, *Oikopleura fusiformis* Fol, 1872, and *Fritillaria borealis* Lohmann, 1896 (Lopez‐Urrutia et al., 2005). Although all species tolerate the range of temperatures and salinities observed in the North Sea (Lopez‐Urrutia et al., 2005), *O. dioica* dominates the other species at temperatures below 20°C (Lombard et al., 2010).

Plankton communities shape the seascape for higher trophic levels (Bertrand et al., 2014) and are considered sentinels of ocean changes (Barton et al., 2013; Benedetti et al., 2021; Cael et al., 2021; Drago et al., 2022), which makes it an important task to understand and monitor their spatio‐temporal variation (Friedland et al., 2020; Hays et al., 2005; McGill et al., 2006; McGinty et al., 2018) and their responses to changing environmental conditions.

Here, we map plankton communities to physically distinct habitats using a fully automated method applied to a high‐frequency in‐situ dataset sampled in the North Sea. Our approach takes advantage of machine learning speed to pry information from a wealth of data and makes the information therein accessible to human researchers. Using a fully connected Autoencoder (AE) and a density‐based clustering algorithm we generate habitat maps from physical and biological variables, sampled with a Remotely Operated Towed Vehicle (ROTV) and investigate plankton–habitat interactions to reveal driving factors behind the compositions of the local plankton communities.

## 2 MATERIALS AND METHODS

### 2.1 Data acquisition and preparation

**FIGURE 1:**
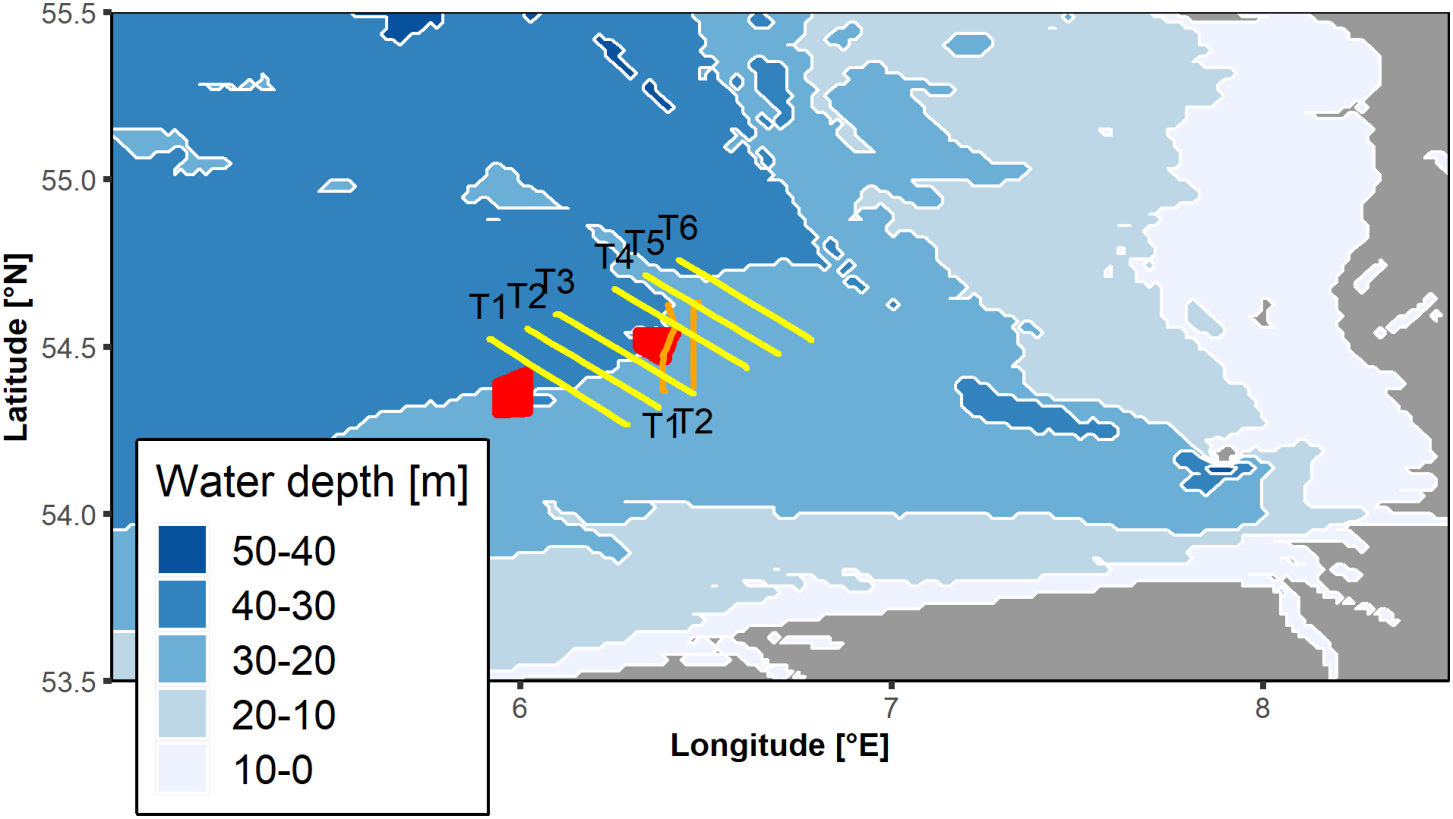
Sampling transects from HE466 (T1–T6, yellow) and HE446 (T1–T2, orange). Red polygons: Wind turbines. Water depth ranges from 0–10 m (white) to 40–50 m (dark blue). A map of the cruises HE429 and HE534 was provided in Plonus, Vogl, and Floeter (2021).

Physical and biological oceanographic measurements were recorded on different North Sea surveys with the RV Heincke (Knust et al., 2017) using a MacArtney TRIAXUS ROTV, complemented by a video plankton recorder (VPR). The TRIAXUS was towed behind the research vessel in an undulating fashion between the sea surface and bottom. A detailed description of the TRIAXUS and the associated sampling procedure can be found in Plonus, Conradt, et al. (2021) and Plonus, Vogl, and Floeter (2021). General data handling was accomplished with R 4.4.0 (RCoreTeam, 2020) and the tidyverse packages “purrr”, “tibble”, “dplyr”, “ggplot2”, “rstatix”, and “tidyr” (Wickham et al., 2019). The map was generated using ‘ggplot2’ and “marmap” (Pante & Simon‐Bouhet, 2013) in R (Figure 1). For our analyses, we selected the following parameters to determine bio‐physical niches in the pelagic realm: temperature (°C), salinity (psu), oxygen (μmol L^−1^), and chlorophyll‐a (RFU). Furthermore, we had sufficient VPR‐derived density data (N L^−1^) available for the plankton groups “Appendicularia”, “Copepoda”, “Dinoflagellates”, “Marine snow”, and “Pluteus larvae”.

Transect diagrams were generated using Ocean Data View (ODV, Schlitzer, 2020) with the embedded spatial interpolation software DIVA (Troupin et al., 2012) and exported as grids with a resolution of ~25 m length × 1 m depth. Abiotic measurements were normalized and rescaled to range from −1 to 1. All plankton density values and chlorophyll a measurements were transformed using the natural logarithm of *x* + 1. The Euclidean distances in the multidimensional space defined by the plankton densities and the chlorophyll a concentration between each grid cell and the top left grid cell of each transect were calculated (Harris et al., 2020). Those distances were normalized and rescaled as described above. This was necessary since deep learning models generally perform better with homogeneous, small values (Bishop, 1995).

The exported grids for each selected parameter and the above‐calculated distance measure were stacked and transformed into feature‐vectors where each grid cell became one vector with 4 features (1 parameter = 1 feature). In our definition, a pelagic micro‐habitat with a spatial extent of ~25 m × 1 m corresponds to one of those feature‐vectors, which reflect the niche space at this point (Colwell & Rangel, 2009).

Based on these feature‐vectors, the AE was trained to reconstruct the original microhabitats and thereby learn relevant abstractions that represent important patterns in the pelagic environment. We used a GPU‐supported Tensorflow backend (Abadi et al., 2015) for Keras (Chollet, 2015) under Python 3.7 (Van Rossum & Drake, 2009) to build and train our AE.

### 2.2 Model description

The AE consisted of four fully connected layers in the Encoder and Decoder, respectively. The Decoder used the transposed weights of the Encoder in reversed order, e.g., the weights of the first Encoder‐Layer were shared with the last Decoder‐Layer. The first layer of the Encoder inflated the 4‐dimensional feature‐vector to a 200‐dimensional feature‐vector, which was reduced to a 100‐, 50‐, and 4‐dimensional feature‐vector by the following layers (4–200–100–50–4). The Decoder did the same in reverse (4–50–100–200–4). The batchsize (number of input microhabitats that are processed simultaneously) was set to 100 and the learning rate followed a sawtooth‐like undulating scheme, initialized at 5e^−8^. Each input feature‐vector corresponded to one microhabitat and included one measurement of each parameter. The model was trained using data recorded during the research cruises HE429 (July 2014) and HE534 (June 2019). The best performance was achieved using a limited training set of only 50.000 randomly selected micro‐habitats over 250 epochs while reserving the remaining ~200.000 microhabitats for validation. During one Epoch each micro‐habitat is presented to the model exactly once.

### 2.3 Microhabitat segregation

The final model was tested using data generated on the research cruise HE466 (June 2016). Compared to the human‐validated plankton densities from HE429 and HE534, the plankton densities for HE466 were estimated exclusively in an automated manner using the threshold procedure proposed by Faillettaz et al. (2016). By applying the trained Encoder only, we created 4‐dimensional representations of the original input. In the following, we will refer to the processing of the micro‐habitats by the Encoder as “projection”. Microhabitats with similar characteristics were projected closer to each other than micro‐habitats with different characteristics. HDBSCAN calculates the Euclidean distance to build clusters from the, in this case, 4‐dimensional inputs (McInnes et al., 2017). We refer to the clustered microhabitats as macrohabitat (MH). MHs were labeled manually. For more information regarding the clustering, see Plonus, Vogl, and Floeter (2021).

### 2.4 Identification of key parameters

While very deep architectures can easily become some kind of “black box”, it was still possible to trace individual inputs in our relatively shallow model which on top consisted only of fully connected Dense‐Layers and did not rely on convolutional filters. Fully connected layers take an n‐dimensional input which is multiplied by a pre‐defined number “X” of weight sets of length n, resulting in a weight matrix of *n* × X weights. Basically, the output of a Dense‐Layer is the dot product of the input and the weight matrix fed into some nonlinear activation function, and thus the weights ultimately define the relative influence of a given input upon the final output. Weights close to zero results in minor changes in the output even if the input variable varies a lot while increasing weights (positive as well as negative) can facilitate major changes in the output. Thus, analyzing the final weights after completion of the training phase revealed the relative influence each input variable had upon a specific output dimension of the Encoder, regardless of the analyzed transect. A similar approach has been used in Drago et al. (2022) to evaluate the importance of single nodes in a random forest algorithm.

### 2.5 Sensitivity analysis

Sensitivity analysis (SA) estimates the importance of an input variable for the model output. We applied a global SA following Sobol (2001) to estimate the importance of each output dimension (D1–D4) of our AE. In a global SE, all parameters are varied simultaneously, allowing not only the contributions of individual parameters to be assessed but also the contribution of their interactions to the variability of the model output.

HDBSCANs prediction method performed poorly on the randomly generated inputs for the SA, likely due to the “curse of dimensionality” (Bellman & Dreyfus, 2015). Randomly generated points were unlikely to be close to HDBSCAN's pre‐estimated “core points” and were therefore usually classified as outliers. Thus, we trained a support vector machine (SVM) to separate the clusters identified by HDBSCAN and to predict the randomly alternating data for the SA. SVMs define boundaries between existing clusters by maximizing the distance between the boundary and each adjacent cluster. Because HDBSCAN forms clusters from spatially distinct groups, SVMs are uniquely qualified to assign randomly generated data points to the closest cluster despite the “curse of dimensionality”. The SVM was trained with the e1071‐package in R (Meyer et al., 2023) and the SA was performed with the SALib‐library in Python (Iwanaga et al., 2022).

### 2.6 Plankton–habitat associations

Plankton–habitat associations were investigated with the R‐package “shar” (Hesselbarth, 2021) which is based on the methods in Plotkin et al. (2000) and Harms et al. (2001). Basically, the expected abundance of a given species is estimated based on bootstrapped randomized habitat maps. Above (or below) a certain threshold a positive (or negative) association between the habitat and the species is assumed. We used 100 randomized habitat maps for each of the original habitat maps and a significance level of 0.01 for the analysis. For comparability, we used the same 100 randomized habitat maps for each of the three investigated groups, namely “Appendicularia”, “Copepods”, and “Pluteus”. We reduced the resolution of our original feature‐vectors (25 m × 1×) to 100 m × 1 m due to computational costs.

## 3 RESULTS

### 3.1 Model training

**FIGURE 2:**
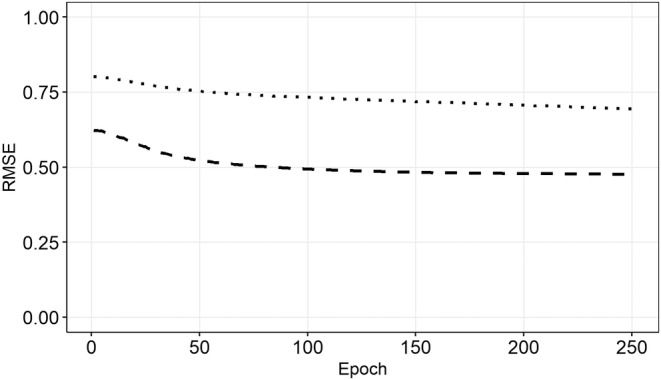
History of the model training. Root‐mean‐squared error (RMSE—dotted) and validation RMSE (dashed).

Each training epoch took ~5 s using a graphic card with 768 gpu‐cores and we trained the model for 250 epochs. The final training (Tr) and validation (Val) Root Mean Squared Error (RMSE) of our model were RMSE_Tr_ ~ 0.69 and RMSE_Val_ ~ 0.48 (Figure 2). As the RMSE were summed over the four features which ranged from −1 to 1, this equals a deviation of ~9% during training and ~6% during validation between the reconstructed and original parameters.

### 3.2 Identification of key parameters

Temperature had the highest impact upon the output dimension 1 (D1) with a ~46% share on the final outcome, followed by salinity with ~36% and the Euclidean distance (~17%). Variations in oxygen had likely no effect on D1 since the oxygen components were weighted down to a share of only ~1%. D2 was dominated by the Euclidean distance (~45%). Temperature and salinity had an equal share of ~23% and oxygen had again the least influence but with ~9% more than on D1. D3 was affected by all input variables similarly with shares between 19% and 29%. D4 was most sensible toward salinity (~39%) followed by temperature (~27%), oxygen (~20%), and finally the Euclidean distance (~14%). Temperature was twice as influential on D1 than on any other output dimension. Variations in oxygen had nearly no effect in D1 but increasingly so on D2, D4, and D3. Overall, salinity was the most influential variable but was never as dominant as temperature or Euclidean distance on a single output dimension. Similar to temperature on D1, the Euclidean distance was twice as influential on D2 as on any other output dimension (Table 1).

**TABLE 1:** Influence of each input variable upon an output variable.

Dimension/variable	Temperature	Oxygen	Salinity	Euclidean
D1	0.462	0.007	0.359	0.171
D2	0.234	0.091	0.225	0.449
D3	0.194	0.288	0.291	0.228
D4	0.273	0.197	0.386	0.144

*Note*: Output variables (dimensions) in rows and input variables in columns.

### 3.3 Sensitivity

**FIGURE 3:**
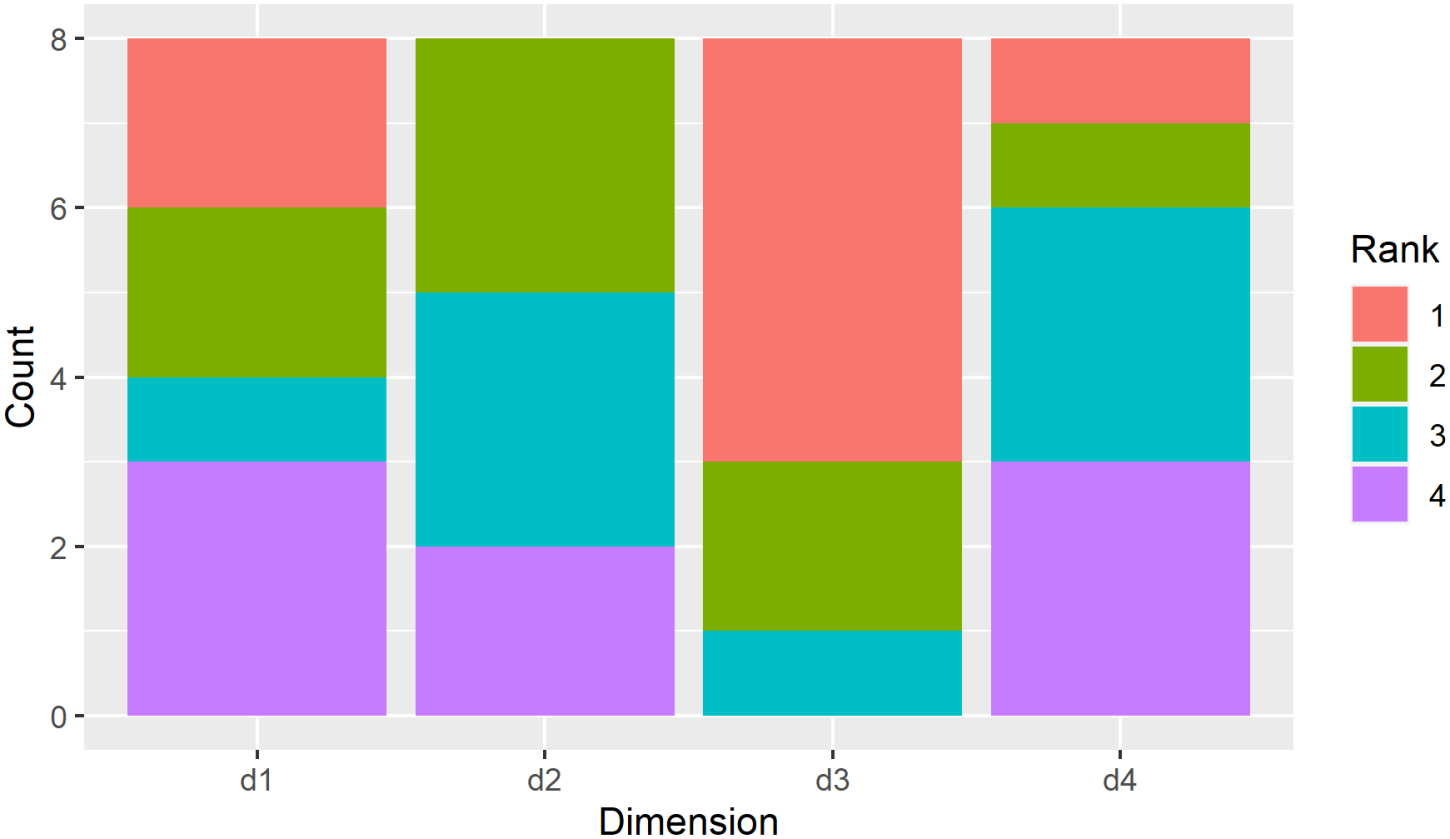
Count of Sobol sensitivity rankings for all transects by dimension. Fixing the dimension with the highest rank (1) would result in the greatest average reduction in output variability.

The most sensitive dimension was D3 in more than half of the cases (Figure 3). D3 was also the most balanced output dimension regarding the influence of different input parameters. The other dimensions were equally important, with D2 never being the most sensitive dimension.

### 3.4 Characterization of macro‐habitats

**FIGURE 4:**
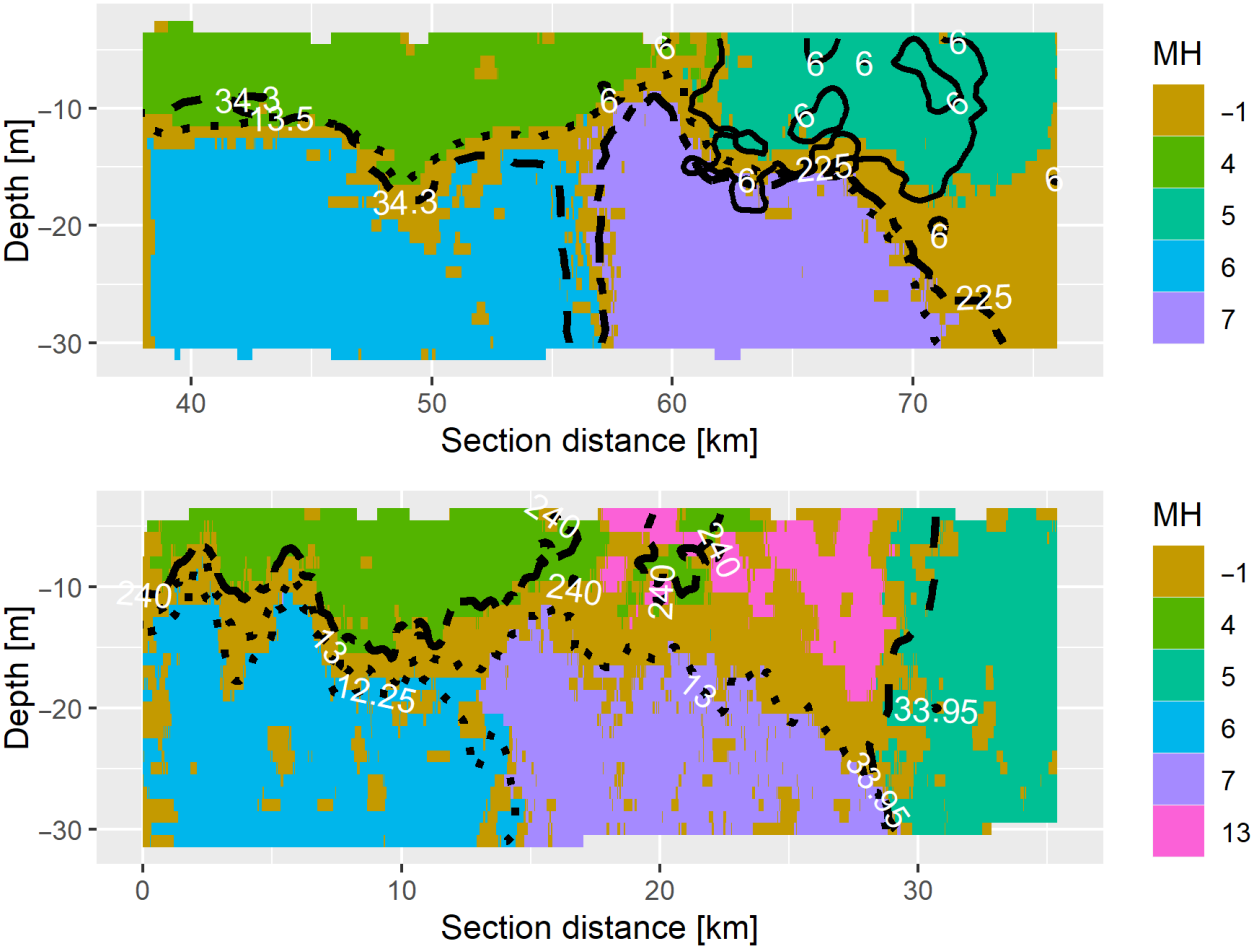
Habitat maps for T1 and T5 of haul 5 from HE466. Dotted: Temperature isolines [°C], dashed: Salinity isolines [psu], dot‐dashed: Oxygen isolines [μmol L^−1^], solid: Pluteus larvae abundance [ind/micro‐habitat]. Each global macro‐habitat is represented by a different color. Brown areas denote the transition areas (originally labeled −1).

The habitat map of HE466 T5 (Figure 4) features everything one could expect from a tidal mixing front as it was described in Hill et al. (1993). At the beginning of the transect is a stratified water column. The surface layer (MH4) is outlined by the 240 μmol L^−1^ isoline for oxygen, with higher concentrations within the MH. The bottom layer (MH6) is characterized by temperatures below 12.25°C. MH7 probably resembles the area of density‐driven circulation and is framed by the 13°C isoline for temperature. Toward the end of the transect (MH5), there is a fully mixed water column with salinity below 33.95 psu. The area left out by those isolines belongs to MH13, which shows the typical characteristics of an along front jet, namely reduced temperatures and upward doming of bottom front isolines.

**FIGURE 5:**
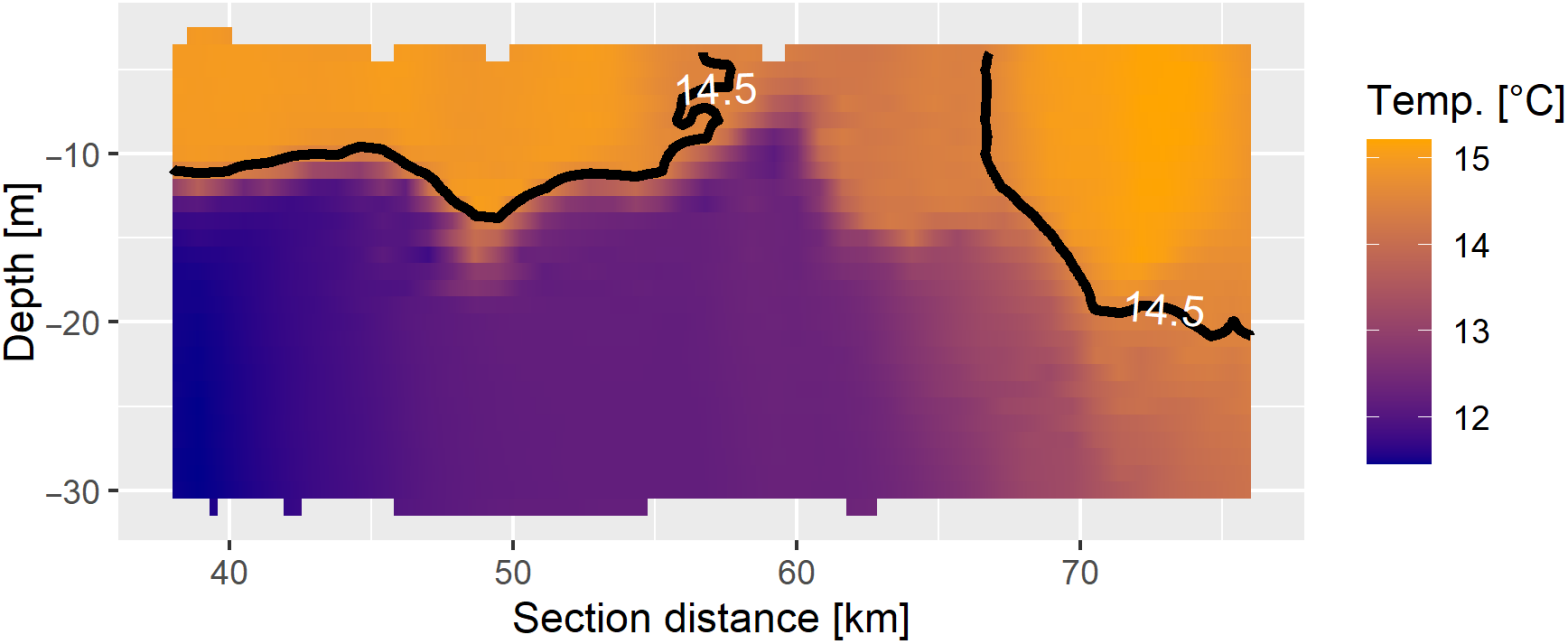
Map of temperature for HE466 haul 5 T1. The black line is the 14.5°C temperature isoline.

At the beginning of the transect T1 is a stratified water column with the surface layer this time limited to temperatures above 13.5°C (MH4). The downwelling pole (MH6) is outlined by the 34.3 psu salinity isoline, with higher salinities within the MH. In contrast, the upwelling pole (MH7) is characterized by oxygen concentrations below 225 μmol L^−1^. There is no fully mixed water column toward the end of the transect, however, what is clustered into MH5 has mostly pluteus larvae densities above 6 individuals per micro‐habitat (ind mh^−1^). The presence of an along front jet as in T5 (MH 13) could explain the accumulation of particles observed in MH5. However, the temperatures do not fully support this (Figure 5). Although it is possible that the transect did not fully cross the tidal mixing front and this could affect the predictive ability of the model, T1 also shows strong evidence of an upwelling–downwelling dipole (Floeter et al., 2022), with the upwelling pole aligned closely with the surface temperature minimum.

As can be seen in Figure 4, the boundaries of global macro‐habitats tend to fit the observed parameter isolines very well. It is noticeable that different parameters contribute to the respective isolines, in T1 there is even an MH that is most closely related to the increased abundance of pluteus larvae.

### 3.5 Plankton–habitat associations

The plankton–habitat associations for all groups and habitats are presented in Table S1. The group “appendicularia” had no general positive or negative association with any of the MHs that occurred in four or more transects. There was a negative association for the groups “pluteus” and “copepods” with MH6 and MH7, which represent the stratified waters of the deeper North Sea below the thermocline. The analysis indicated a generally positive association with the surface mixed layer and the fully mixed coastal area for both groups as well.

**FIGURE 6:**
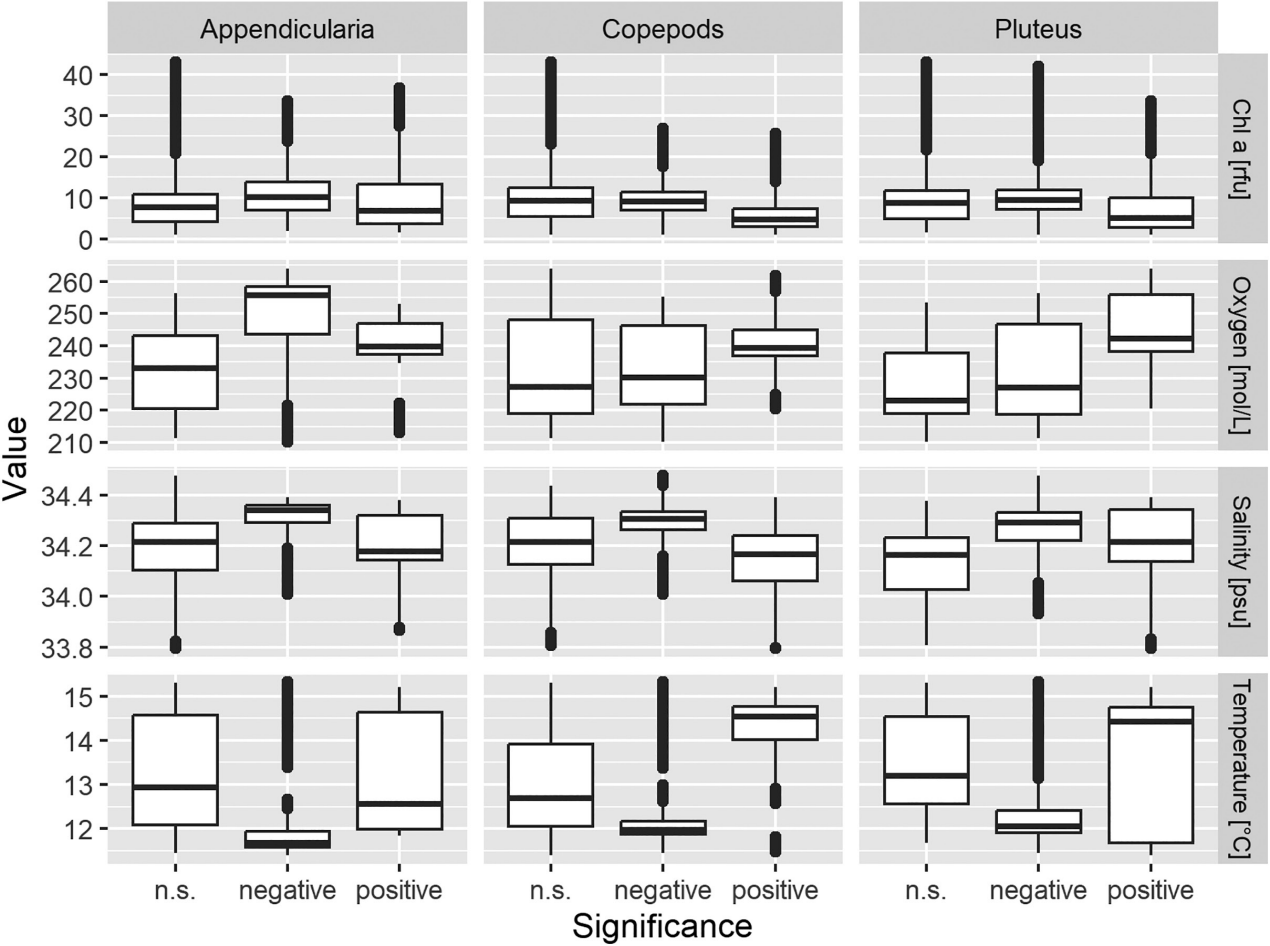
Overview for temperature [°C], salinity [psu], oxygen [mol/L], and chlorophyll a [rfu] in areas with a positive, negative, or not significant (n.s.) association with Appendicularia, Copepods, or Pluteus larvae. Positive associations indicate an increased abundance of the respective group in the underlying area and therefore the parameter ranges indicate the optimal niche space for that plankton group.

Parameter ranges of MHs with a positive association with any specific group of organisms did not indicate a distinct physical niche (Figure 6). An exception was the positive association of copepods with MHs of higher temperatures and a negative association with MHs of lower temperatures. Furthermore, the analysis indicated an affinity of appendicularia toward the areas of lower oxygen concentrations.

## 4 DISCUSSION

### 4.1 Model inputs

The model had difficulty interpreting plankton abundance, most likely due to the skewness of the data. The patchiness of plankton produces many zeros and only relatively few high abundances. Using the Euclidean distance had two main advantages. First, the distance was no longer biased toward zero. Secondly, the Euclidea distance combined the data on the resident plankton communities, using chlorophyll a as a proxy for phytoplankton abundance, into one variable. In theory, the model can be trained with any number of species, groups, or even functional groups, if applied to data that allows such distinctions.

Furthermore, the feature vectors we use describe the Eltonian niche space (Dehling & Stouffer, 2018) at a specific location and the final macro‐habitat is therefore basically a cluster of locations with very similar niche spaces. This makes our method uniquely qualified to study species‐habitat interactions and ecological niches, which is of utmost importance considering the ongoing climatic changes (Benedetti et al., 2021; Busseni et al., 2020; Cael et al., 2021). This would require a more detailed dataset than the one used in the current investigation, but there are advantages in using a less detailed dataset as applied in this study. The fully automated data pipeline makes it possible to get an initial near real‐time assessment of the pelagic habitats and associated species communities currently present in the study area and therefore helps monitor ongoing changes.

### 4.2 Biophysical characteristics of macro‐habitats

Transects T1 to T6 of HE466 (Figures S1–S6) were not only in the immediate vicinity of an offshore wind farm (OWF) but partly also crossed a tidal mixing front (Hill et al., 1993). While in T1–T3 all MHs had mean salinities >34 psu, there was a decreasing trend in salinity in the transects T4–T6, indicating a shift from North Sea to coastal water (Lee, 1980). Only T5 had an MH that spanned the entire water column with a mean salinity <34 psu, indicating a fully mixed habitat with characteristics of coastal water. T4 and T6 did not extend into the mixed coastal water.

In general, the model produced a vertical segregation along the thermocline. That is already an important feature since the mixed‐layer depth is a good indicator of the productivity and biodiversity of plankton communities (Barton et al., 2013; Brun et al., 2015). Especially the distinct upwelling‐downwelling pattern of the dipole in T1 of HE466 shows the ability of our model to accurately track the surface mixed layer.

The horizontal segregations are probably related to changing velocities. Those also induce some small changes in the physical characteristics, which is what our model was able to detect. The velocity patterns of dipoles (Broström, 2008) and fronts (Hill et al., 1993) are well described in the literature and closely match the habitat maps we generated.

### 4.3 Plankton niches

On a global scale machine learning has successfully been applied to reevaluate existing datasets and link plankton communities to distinct physical habitats (Busseni et al., 2020; Drago et al., 2022; Panaïotis et al., 2023; Sonnewald et al., 2020), thereby defining what has been called ecological provinces (Sonnewald et al., 2020). Especially the data from the CPR survey has frequently been used to additionally show the changing distribution patterns of a variety of planktic species in response to climate change (Barton et al., 2013; Beaugrand et al., 2009; Benedetti et al., 2021). The data used in this study covers the much smaller sub‐mesoscale, where imaging and machine learning can help to improve spatial resolution (Irisson et al., 2022). Our analysis did not indicate niche segregation between the three studied plankton groups, since multiple groups were regularly found within the same MH. Furthermore, the MHs positively associated with specific groups showed no distinctly different physical properties from other areas. There are several potential reasons for the lack of readily observable niches. The lack of taxonomic details could mask the segregation of morphologically similar species of any of the groups into distinct niches. For example, the calanoid copepods *Calanus finmarchicus* Gunnerus, 1770 and *Calanus helgolandicus* Claus, 1863 both contributed significantly to copepod biomass during summer (Jónasdóttir & Koski, 2011). However, the cold‐boreal *C. finmarchicus* (Planque & Fromentin, 1996) was restricted to the cooler bottom waters while *C. helgolandicus* generally prefers warmer waters (Planque & Fromentin, 1996) and occupied the upper surface‐mixed layer. The preferred temperatures for *C. finmarchicus* of <9°C (Jónasdóttir & Koski, 2011) were clearly exceeded in the present sampling area (>12°C) and considering the general northward drift of plankton distributions (Barton et al., 2013; Cael et al., 2021; Martin et al., 2021), and of *C. finmarchicus* specifically (Wilson et al., 2016), it is unlikely that this particular example caused the negative findings in this study. In general, we think it reasonable that despite the great biodiversity of the North Sea, it is possible to investigate spatial niches using VPR images since, as we argued in our introduction, the different taxa and communities are usually dominated by specific species.

Prey selection is another possible mechanism for niche diversity (Cleary et al., 2016; Fransz et al., 1991), which is not revealed using VPR images or images at all. This however would also require different species to be very abundant to mask any possible findings on the sub‐mesoscale. Of course, there is the possibility that niche segregation does occur for the less common species, but those processes probably act on even smaller scales (Basterretxea et al., 2020; Mitchell et al., 2008) and are not in the scope of this investigation.

Next to temperature, which is frequently identified as a major force behind plankton distribution patterns (Busseni et al., 2020; Lindegren et al., 2020; Wilson et al., 2016; e.g. Benedetti et al., 2021; Drago et al., 2022; Houliez et al., 2021), the local hydrography is a primary driver of plankton distributions and biodiversity (Beaugrand et al., 2001; Beaugrand & Ibañez, 2002; Sonnewald et al., 2020; Swalethorp et al., 2015). Since plankton is, per definition, subjected to the currents that shape the pelagic environment, the strong velocities associated with upwelling‐downwelling dipoles (Broström, 2008) and frontal jets (Hill et al., 1993) have the potential to superimpose behavioral niche segregation (Hidalgo et al., 2012). We believe that this is the most likely explanation for the lack of physically distinct niches in our relatively short transects. A similar result was observed with an Underwater Vision Profiler 6 (Panaïotis et al., 2024) and by examining a picophytoplankton dataset using machine learning (Chen et al., 2020).

In areas less dominated by strong currents like the vast, oligotrophic parts of the Atlantic and Pacific Ocean, machine learning approaches as presented in this study can improve our understanding of how physical gradients shape plankton communities (Chen et al., 2020; Greer et al., 2023). And optical datasets are especially useful in investigating the understudied gelatinous grazers (Greer et al., 2023), which will likely become more important under increasing temperatures (Winder et al., 2017). Thus, optical sampling methods and machine learning will be important tools to investigate the changes in our oceans.

## AUTHOR CONTRIBUTIONS

**Rene‐Marcel Plonus:** Formal analysis (lead); methodology (equal); writing – original draft (lead); writing – review and editing (equal). **Jens Floeter:** Methodology (supporting); resources (lead); supervision (lead); writing – review and editing (equal).

## FUNDING INFORMATION

Alfred Wegener Institut Helmholtz Centre for Polar and Marine Research were supported by grant numbers AWI_HE429_00, AWI_HE446_00, AWI_HE466_00, and AWI_HE534_00.

## CONFLICT OF INTEREST STATEMENT

The authors declare that the research was conducted in the absence of any commercial or financial relationships that could be construed as a potential conflict of interest.

## Supporting information


Data S1.

## Data Availability

The data that support the findings of this study are openly available in Dryad at https://doi.org/10.5061/dryad.34tmpg4s4.
