# Supplementary material for: Identification of plankton habitats in the North Sea
Source: Ecol Evol. 2024 Sep 30;14(10):e70342. doi: 10.1002/ece3.70342 (PMC11440368; doi:10.1002/ece3.70342)
Supplement: Supplementary file 1 — Data S1. [file ECE3-14-e70342-s001.docx]

**Identification of plankton habitats in the North Sea**

Rene-Marcel Plonus*, Jens Floeter

Keywords: habitat maps, machine learning, North Sea, plankton distributions, plankton-habitat-associations

*Correspondence: [rene-marcel.plonus@uni-hamburg.de](mailto:rene-marcel.plonus@uni-hamburg.de)

Institute of marine ecosystem and fishery science, University of Hamburg, Hamburg, Germany.

This document refers to the paper ‘Identification of plankton habitats in the North Sea’ published by the above-mentioned authors in ‘Ecology and Evolution’. It provides detailed information about the investigated species-habitat associations and an exemplary overview for the physical parameters measured during HE466 using the ROTV Triaxus, visualized with Ocean Data View.

# Supplementary Material

## Tables

Supplementary Table S 1: Results for plankton-habitat associations based on the R-package shar. ‘lo’ and ‘hi’ give the boundaries for ‘count’. Positive or negative associations between a species and the habitat occur if ‘count’ is below ‘lo’ or above ‘hi’. n.s.: not significant.

| **cruise** | **taxon group** | **habitat** | **count** | **lo** | **hi** | **statistically significant** |
| --- | --- | --- | --- | --- | --- | --- |
| HE446_H06T1 | Appendicularia | 1 | 1386 | 1756 | 3113 | negative |
| HE446_H06T1 | Appendicularia | 2 | 1207 | 366 | 1401 | n.s. |
| HE446_H06T1 | Appendicularia | 3 | 2259 | 1014 | 2287 | n.s. |
| HE446_H06T1 | Pluteus | 1 | 292641 | 122746 | 247726 | positive |
| HE446_H06T1 | Pluteus | 2 | 32117 | 32349 | 120161 | negative |
| HE446_H06T1 | Pluteus | 3 | 72868 | 84711 | 209973 | negative |
| HE446_H06T1 | Copepods | 1 | 9846 | 5376 | 9342 | positive |
| HE446_H06T1 | Copepods | 2 | 2364 | 1210 | 3904 | n.s. |
| HE446_H06T1 | Copepods | 3 | 3431 | 4142 | 7580 | negative |
| HE446_H06T2 | Appendicularia | 1 | 1719 | 2428 | 5502 | negative |
| HE446_H06T2 | Appendicularia | 3 | 4418 | 635 | 3709 | positive |
| HE446_H06T2 | Pluteus | 1 | 426316 | 267016 | 393514 | positive |
| HE446_H06T2 | Pluteus | 3 | 62343 | 95145 | 221643 | negative |
| HE446_H06T2 | Copepods | 1 | 17202 | 15192 | 18522 | n.s. |
| HE446_H06T2 | Copepods | 3 | 6980 | 5660 | 8990 | n.s. |
| HE466_H05T1 | Appendicularia | 4 | 2460 | 2301 | 8058 | n.s. |
| HE466_H05T1 | Appendicularia | 5 | 9398 | 1731 | 7407 | positive |
| HE466_H05T1 | Appendicularia | 6 | 5088 | 4708 | 10532 | n.s. |
| HE466_H05T1 | Appendicularia | 7 | 4245 | 3209 | 9370 | n.s. |
| HE466_H05T1 | Pluteus | 4 | 7125 | 1629 | 22761 | n.s. |
| HE466_H05T1 | Pluteus | 5 | 31613 | 1144 | 18630 | positive |
| HE466_H05T1 | Pluteus | 6 | 1128 | 6708 | 28068 | negative |
| HE466_H05T1 | Pluteus | 7 | 4952 | 2560 | 25783 | n.s. |
| HE466_H05T1 | Copepods | 4 | 18613 | 9545 | 52289 | n.s. |
| HE466_H05T1 | Copepods | 5 | 66357 | 7526 | 49377 | positive |
| HE466_H05T1 | Copepods | 6 | 7633 | 30099 | 71050 | negative |
| HE466_H05T1 | Copepods | 7 | 41834 | 14051 | 63788 | n.s. |
| HE466_H05T2 | Appendicularia | 4 | 3247 | 2686 | 5062 | n.s. |
| HE466_H05T2 | Appendicularia | 5 | 3273 | 1406 | 3625 | n.s. |
| HE466_H05T2 | Appendicularia | 6 | 1526 | 1400 | 3481 | n.s. |
| HE466_H05T2 | Appendicularia | 7 | 7383 | 5732 | 8540 | n.s. |
| HE466_H05T2 | Pluteus | 4 | 16954 | 1779 | 13363 | positive |
| HE466_H05T2 | Pluteus | 5 | 5685 | 628 | 10017 | n.s. |
| HE466_H05T2 | Pluteus | 6 | 351 | 448 | 11846 | negative |
| HE466_H05T2 | Pluteus | 7 | 3489 | 7778 | 19784 | negative |
| HE466_H05T2 | Copepods | 4 | 31057 | 19004 | 58469 | n.s. |
| HE466_H05T2 | Copepods | 5 | 45676 | 9151 | 42409 | positive |
| HE466_H05T2 | Copepods | 6 | 2984 | 7994 | 36503 | negative |
| HE466_H05T2 | Copepods | 7 | 64928 | 51745 | 90504 | n.s. |
| HE466_H05T3 | Appendicularia | 4 | 7844 | 6459 | 9188 | n.s. |
| HE466_H05T3 | Appendicularia | 6 | 5208 | 3636 | 6351 | n.s. |
| HE466_H05T3 | Appendicularia | 7 | 2767 | 2319 | 4546 | n.s. |
| HE466_H05T3 | Appendicularia | 8 | 1572 | 744 | 2286 | n.s. |
| HE466_H05T3 | Pluteus | 4 | 37048 | 7990 | 30629 | positive |
| HE466_H05T3 | Pluteus | 6 | 1150 | 3308 | 25539 | negative |
| HE466_H05T3 | Pluteus | 7 | 2729 | 2175 | 23363 | n.s. |
| HE466_H05T3 | Pluteus | 8 | 1355 | 423 | 11661 | n.s. |
| HE466_H05T3 | Copepods | 4 | 72566 | 39565 | 71156 | positive |
| HE466_H05T3 | Copepods | 6 | 14511 | 19974 | 51499 | negative |
| HE466_H05T3 | Copepods | 7 | 20670 | 12639 | 40734 | n.s. |
| HE466_H05T3 | Copepods | 8 | 11596 | 3954 | 20636 | n.s. |
| HE466_H05T4 | Appendicularia | 5 | 6005 | 5333 | 7391 | n.s. |
| HE466_H05T4 | Appendicularia | 6 | 3781 | 1842 | 3993 | n.s. |
| HE466_H05T4 | Appendicularia | 9 | 754 | 629 | 1921 | n.s. |
| HE466_H05T4 | Appendicularia | 11 | 2096 | 1163 | 2508 | n.s. |
| HE466_H05T4 | Appendicularia | 12 | 2360 | 1735 | 3833 | n.s. |
| HE466_H05T4 | Appendicularia | 13 | 1150 | 724 | 1970 | n.s. |
| HE466_H05T4 | Pluteus | 5 | 35808 | 9169 | 25702 | positive |
| HE466_H05T4 | Pluteus | 6 | 679 | 1784 | 18604 | negative |
| HE466_H05T4 | Pluteus | 9 | 1709 | 309 | 11072 | n.s. |
| HE466_H05T4 | Pluteus | 11 | 571 | 998 | 13658 | negative |
| HE466_H05T4 | Pluteus | 12 | 2805 | 1908 | 18310 | n.s. |
| HE466_H05T4 | Pluteus | 13 | 361 | 483 | 10558 | negative |
| HE466_H05T4 | Copepods | 5 | 91784 | 47084 | 78054 | positive |
| HE466_H05T4 | Copepods | 6 | 9786 | 11938 | 43754 | negative |
| HE466_H05T4 | Copepods | 9 | 4617 | 3978 | 24451 | n.s. |
| HE466_H05T4 | Copepods | 11 | 14856 | 8012 | 31071 | n.s. |
| HE466_H05T4 | Copepods | 12 | 23776 | 13543 | 42728 | n.s. |
| HE466_H05T4 | Copepods | 13 | 8731 | 5437 | 24604 | n.s. |
| HE466_H05T5 | Appendicularia | 4 | 3807 | 3470 | 6009 | n.s. |
| HE466_H05T5 | Appendicularia | 5 | 5767 | 3602 | 6283 | n.s. |
| HE466_H05T5 | Appendicularia | 6 | 6206 | 4503 | 7073 | n.s. |
| HE466_H05T5 | Appendicularia | 7 | 5056 | 3954 | 6821 | n.s. |
| HE466_H05T5 | Appendicularia | 14 | 2065 | 1141 | 3309 | n.s. |
| HE466_H05T5 | Pluteus | 4 | 5685 | 1416 | 11744 | n.s. |
| HE466_H05T5 | Pluteus | 5 | 10542 | 1208 | 12173 | n.s. |
| HE466_H05T5 | Pluteus | 6 | 1065 | 3414 | 14038 | negative |
| HE466_H05T5 | Pluteus | 7 | 1610 | 2081 | 14039 | negative |
| HE466_H05T5 | Pluteus | 14 | 9315 | 256 | 8142 | positive |
| HE466_H05T5 | Copepods | 4 | 27692 | 19861 | 41377 | n.s. |
| HE466_H05T5 | Copepods | 5 | 43775 | 21143 | 44226 | n.s. |
| HE466_H05T5 | Copepods | 6 | 31911 | 33556 | 52335 | negative |
| HE466_H05T5 | Copepods | 7 | 31285 | 28944 | 51483 | n.s. |
| HE466_H05T5 | Copepods | 14 | 22963 | 8201 | 22661 | positive |
| HE466_H05T6 | Appendicularia | 4 | 266 | 833 | 2662 | negative |
| HE466_H05T6 | Appendicularia | 5 | 3670 | 1801 | 4139 | n.s. |
| HE466_H05T6 | Appendicularia | 6 | 985 | 1163 | 3492 | negative |
| HE466_H05T6 | Appendicularia | 15 | 867 | 284 | 1918 | n.s. |
| HE466_H05T6 | Appendicularia | 16 | 2725 | 389 | 2242 | positive |
| HE466_H05T6 | Pluteus | 4 | 236 | 985 | 11940 | negative |
| HE466_H05T6 | Pluteus | 5 | 23589 | 3533 | 12773 | positive |
| HE466_H05T6 | Pluteus | 6 | 2926 | 2450 | 13951 | n.s. |
| HE466_H05T6 | Pluteus | 15 | 398 | 231 | 7558 | n.s. |
| HE466_H05T6 | Pluteus | 16 | 1218 | 621 | 10139 | n.s. |
| HE466_H05T6 | Copepods | 4 | 4057 | 6363 | 37198 | negative |
| HE466_H05T6 | Copepods | 5 | 71464 | 19930 | 48791 | positive |
| HE466_H05T6 | Copepods | 6 | 10456 | 14951 | 48632 | negative |
| HE466_H05T6 | Copepods | 15 | 11465 | 3205 | 24135 | n.s. |
| HE466_H05T6 | Copepods | 16 | 16305 | 4106 | 31686 | n.s. |

## Figures


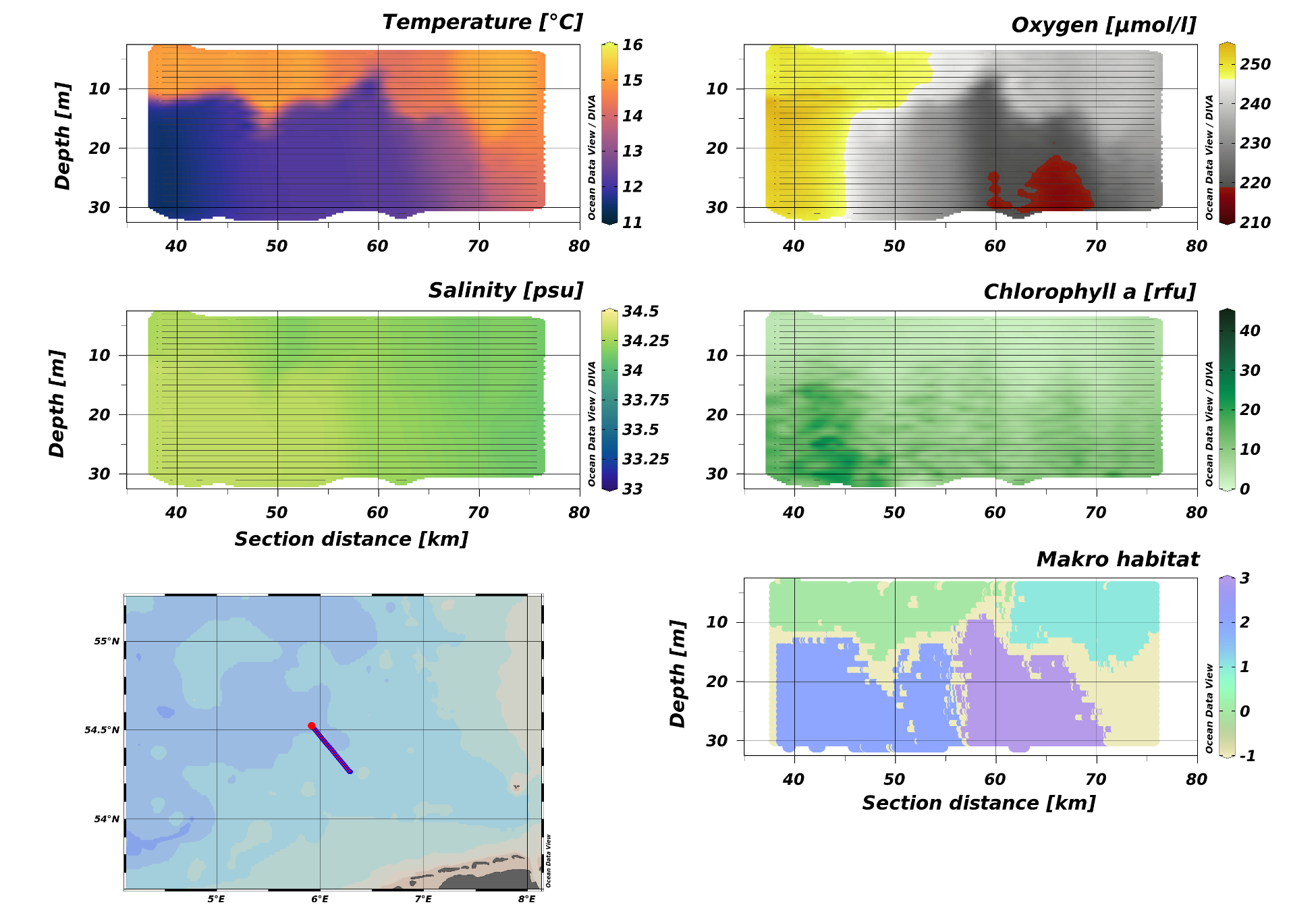


Supplementary Figure S 1: Gridded parameters and habitat map for HE466 T1. The lower left panel shows the location of the transect. The parameter ranges are the same for all transects of HE466.


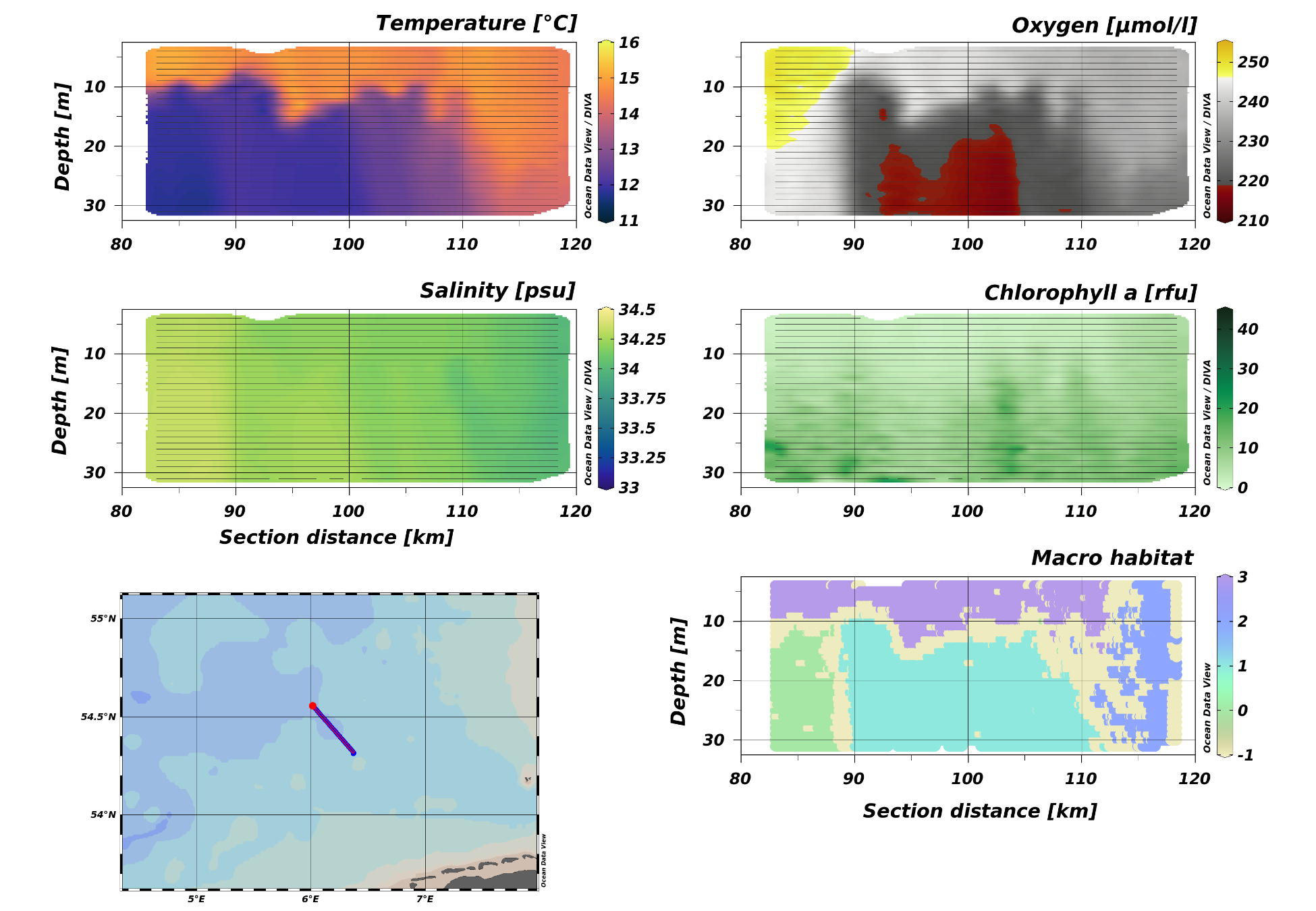


Supplementary Figure S 2: Gridded parameters and habitat map for HE466 T2. The lower left panel shows the location of the transect. The parameter ranges are the same for all transects of HE466.


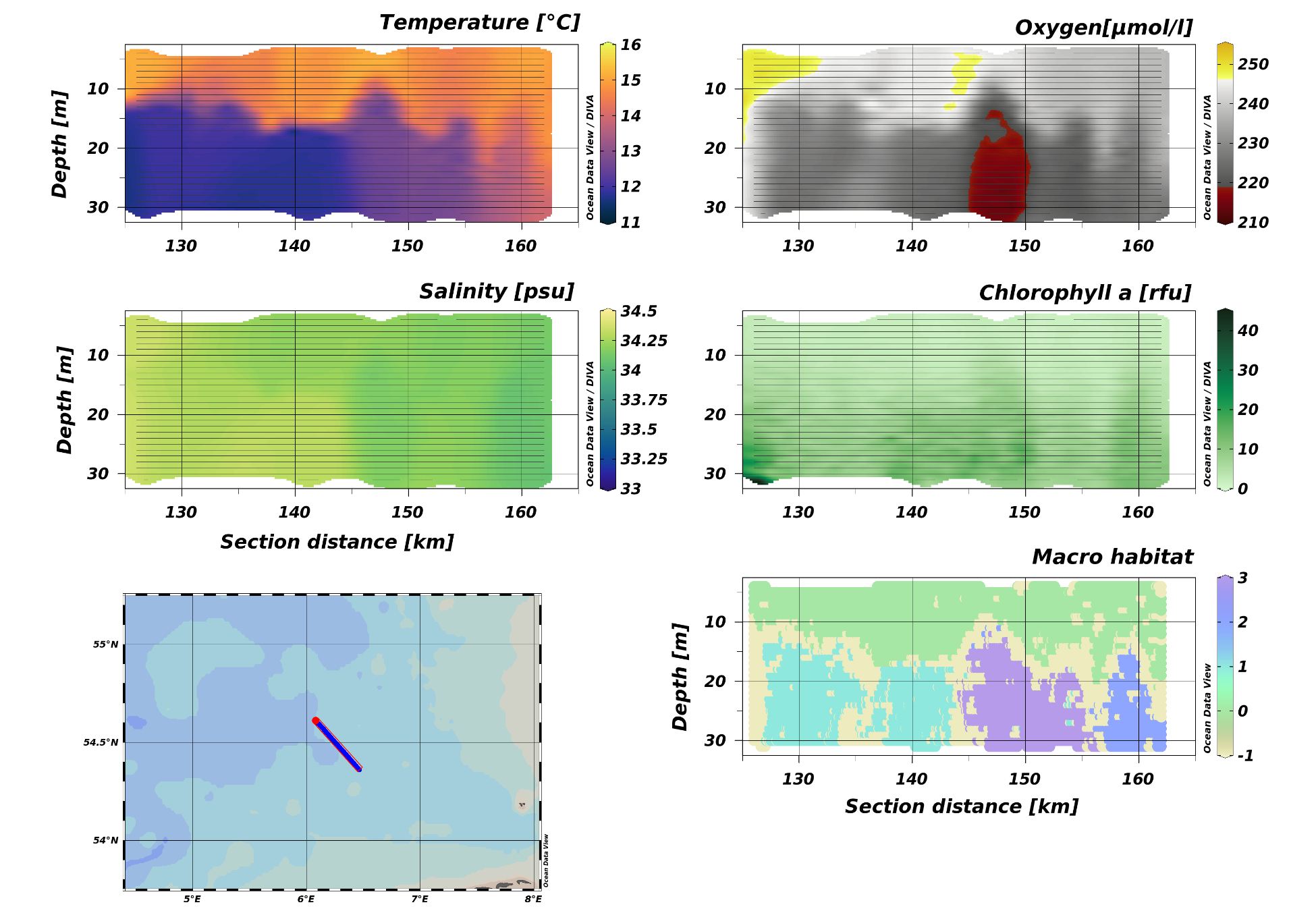


Supplementary Figure S 3: Gridded parameters and habitat map for HE466 T3. The lower left panel shows the location of the transect. The parameter ranges are the same for all transects of HE466.


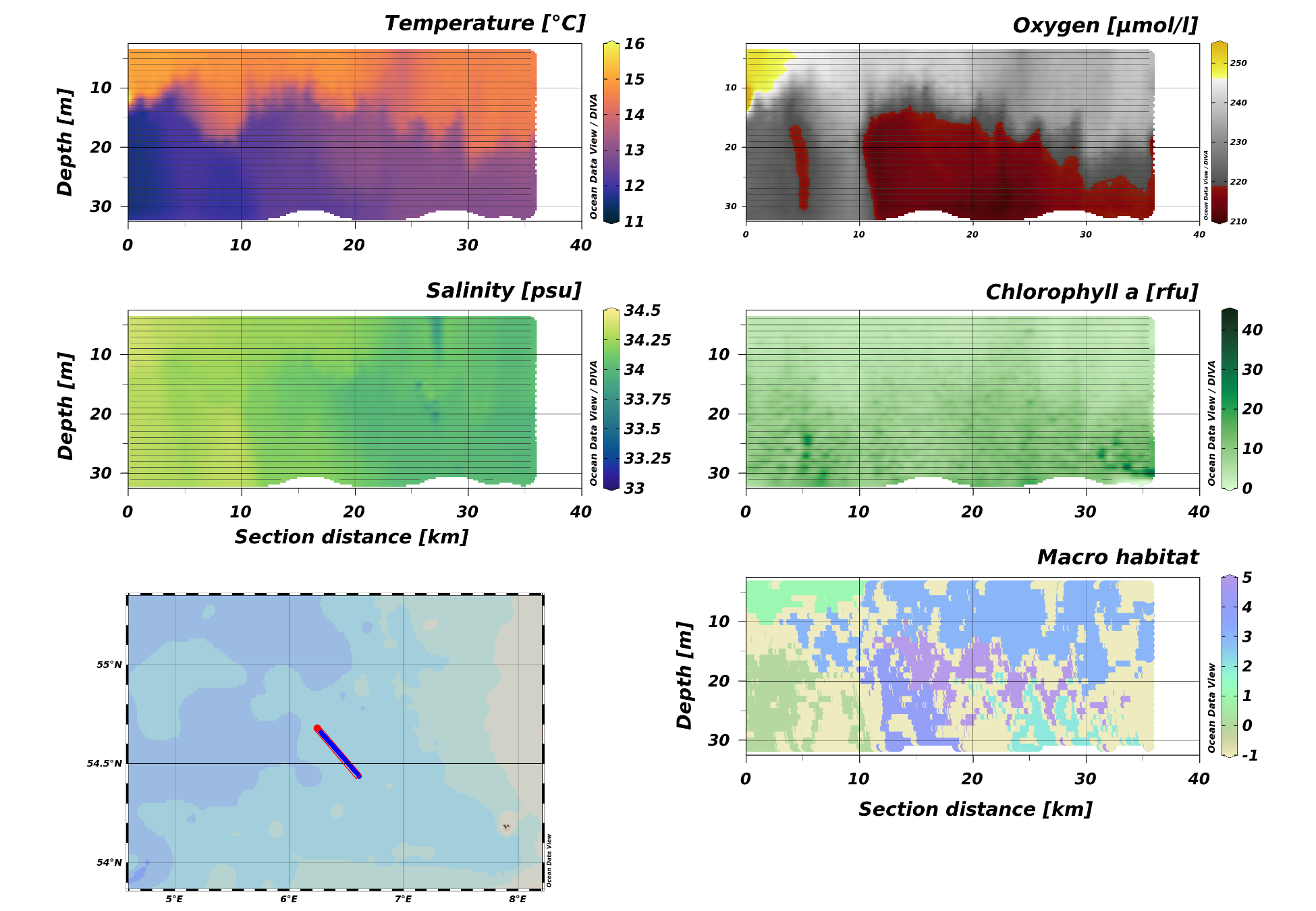


Supplementary Figure S 4: Gridded parameters and habitat map for HE466 T4. The lower left panel shows the location of the transect. The parameter ranges are the same for all transects of HE466.


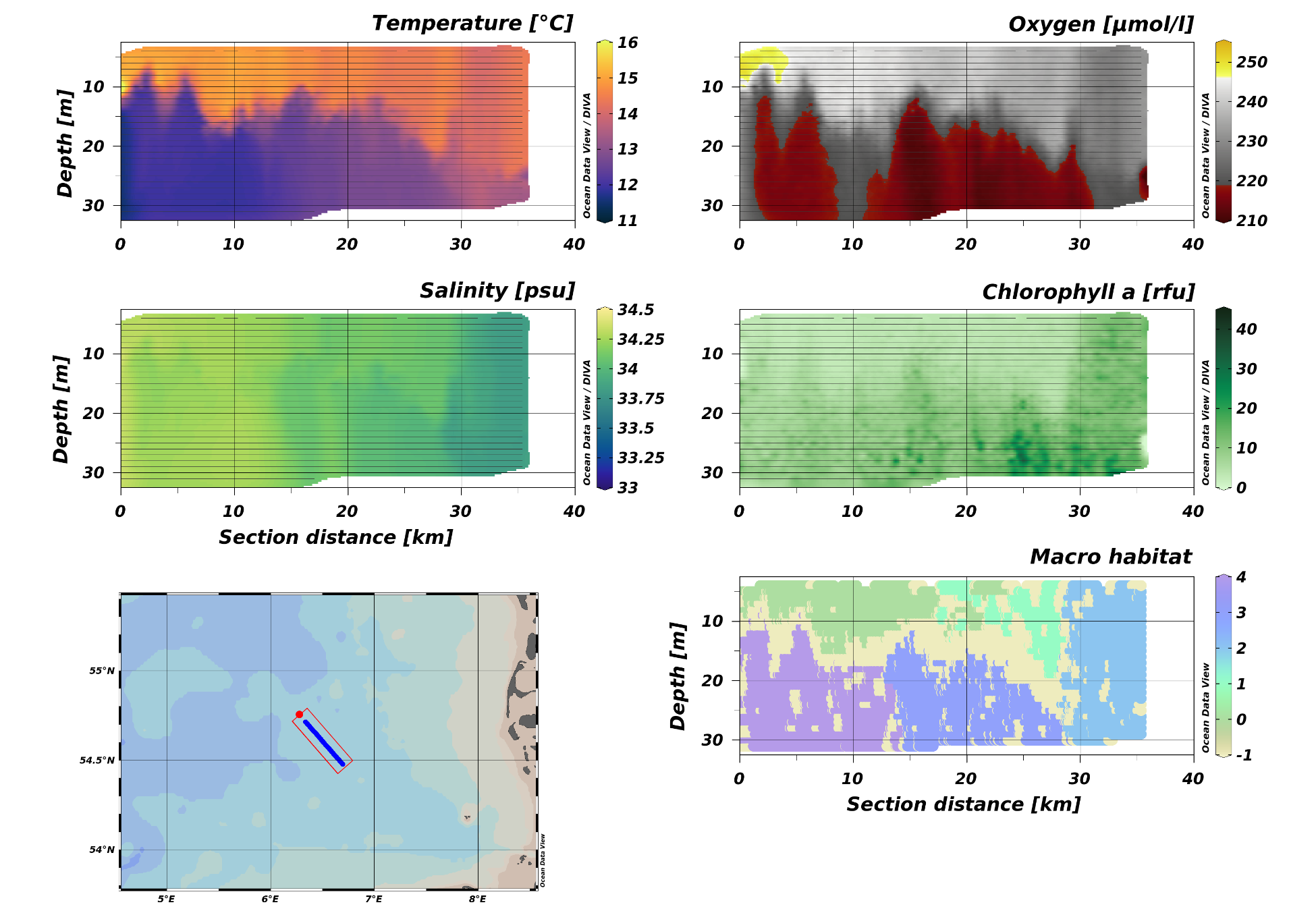


Supplementary Figure S 5: Gridded parameters and habitat map for HE466 T5. The lower left panel shows the location of the transect. The parameter ranges are the same for all transects of HE466.


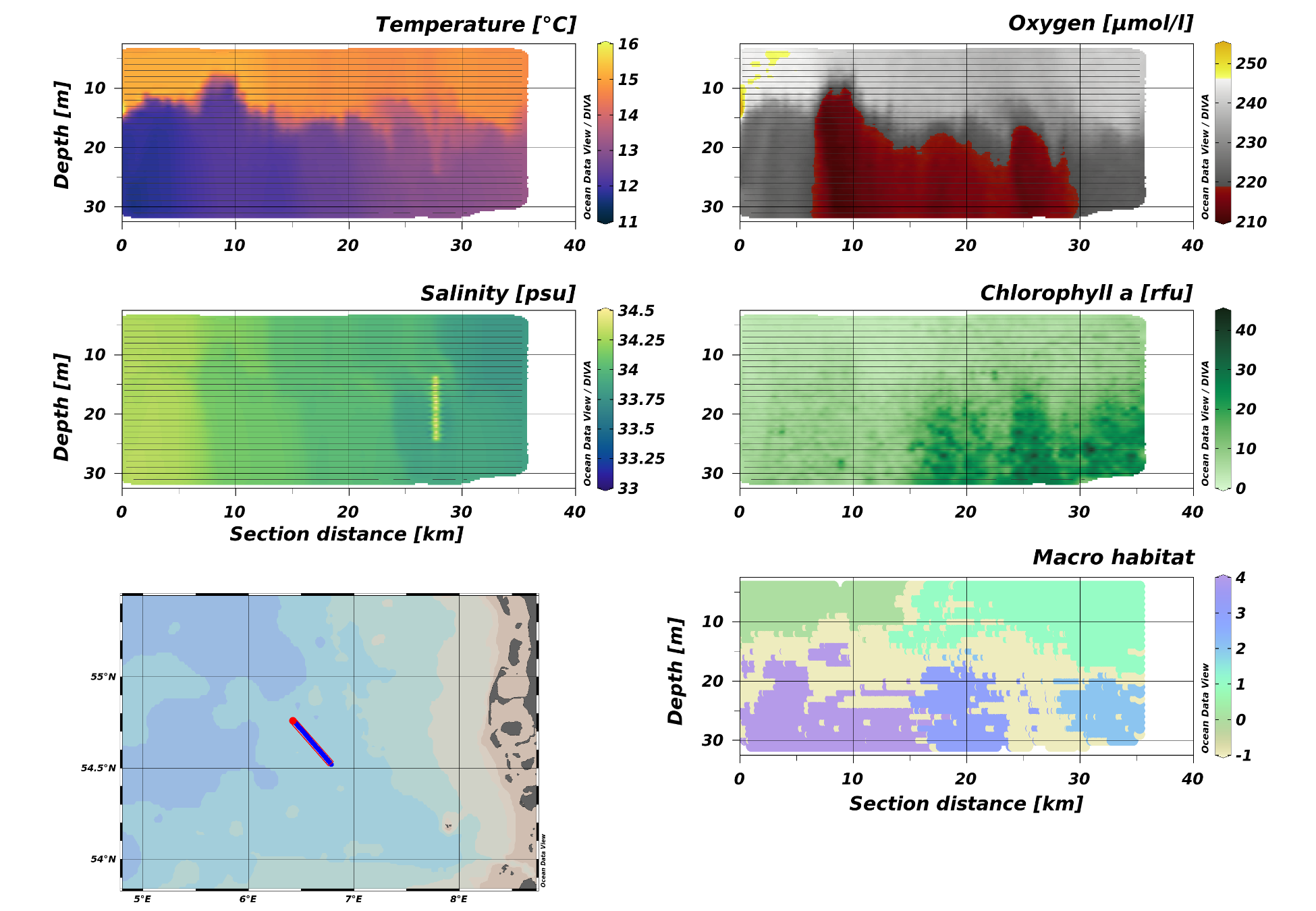


Supplementary Figure S 6: Gridded parameters and habitat map for HE466 T6. The lower left panel shows the location of the transect. The parameter ranges are the same for all transects of HE466.
